# Complete Genome Sequence of Rhizobium phaseoli Podophage Palo

**DOI:** 10.1128/MRA.01443-20

**Published:** 2021-01-28

**Authors:** Ali Nabhani, Leika Rushing, Heather Newkirk, Ben Burrowes, Ryland Young, Carlos Gonzalez

**Affiliations:** a Department of Biochemistry and Biophysics, Texas A&M University, College Station, Texas, USA; b Department of Plant Pathology and Microbiology, Texas A&M University, College Station, Texas, USA; c Center for Phage Technology, Texas A&M University, College Station, Texas, USA; Portland State University

## Abstract

Here, we present the genome of Palo, a T7-like podophage of Rhizobium phaseoli. The genome is 46.3 kb and contains 58 predicted protein-coding genes, including a novel signal-anchor-release (SAR) endolysin, a homolog of the T5 A1 protein required for DNA transfer, and a dual-start holin/antiholin pair.

## ANNOUNCEMENT

The Gram-negative alphaproteobacterium Rhizobium phaseoli is generally found in legume root nodules, where it fixes nitrogen (1). R. phaseoli possesses the *sym* plasmid, containing symbiotic determinants, nodulation genes, and nitrogen fixation gene repeats (2). Examined in this report is the genome of Palo, a T7-like podophage of R. phaseoli that has multiple unique features and could be used to study the population dynamics of R. phaseoli populations in soil.

Bacteriophage Palo was isolated from potato root samples obtained in Olton, Texas, by plaque purification using the double-agar overlay method (3) with host bacterium R. phaseoli 127K17 grown aerobically at 28°C in l-arabinose medium. Genomic DNA was isolated from crude lysates using the modified Wizard cleanup kit protocol, as described previously (4). Samples were then prepared as Illumina TruSeq libraries using a Nextera Flex kit and sequenced on an Illumina MiSeq system using paired-end 250-bp reads and 500-cycle v2 chemistry. The 444,176 reads were quality controlled and manually trimmed using FastQC (www.bioinformatics.babraham.ac.uk/projects/fastqc) and FastX v0.0.14 (http://hannonlab.cshl.edu/fastx_toolkit/download.html), respectively. A single contig was assembled to 22.5-fold coverage using SPAdes v3.5.0 with default parameters (5). The contig was closed by PCR and Sanger sequencing using primers AAGGATTGGTTCGCATCTACC and ATACTGAGTTCGATCCTCTGCA. GLIMMER v3 (6) and MetaGeneAnnotator v1.0 (7) were used for structural annotation of the genome, and tRNAs were detected using ARAGORN v2.36 (8). BLAST v2.9.0 (9) was run against the NCBI nonredundant, TrEMBL, and UniProtKB Swiss-Prot databases (10) (accessed 14 March 2020). InterProScan v5.33 (11) was used to conduct conserved domain searches, and TMHMM v2.0 (12) was used to predict transmembrane domains. All programs were used with default settings. progressiveMauve v2.4 was used to calculate genome-wide nucleotide similarity (13). These annotation tools were used in Apollo and Galaxy instances hosted by the Center for Phage Technology at Texas A&M University (https://cpt.tamu.edu/galaxy-pub) (14–16).

Genomic analysis determined that Palo is a podophage, which was confirmed visually by transmission electron microscopy (TEM), with a 46,322-bp genome and a GC content of 52%, significantly lower than the 61.1% GC content of its host. PhageTerm (17) predicted 260-bp direct terminal repeats, like those characteristic of T7-like phages. The genome has a coding density of 93%, 58 predicted protein-coding genes, and no tRNAs. BLASTn analysis showed that the phage most closely related to Palo was the T7-like phage RHEph09, a virulent Rhizobium etli phage, with an overall nucleotide sequence identity of 54.5% (18). Twenty-four of the 58 putative protein-coding genes of Palo were assigned predicted functions. A putative dual-start holin/antiholin with a predicted mRNA stem-loop that could control translation initiation was found. A putative novel signal-anchor-release (SAR) endolysin gene that possessed not only the SAR domain at the N terminus but also a separate transmembrane domain at the C terminus was identified. Surprisingly, although Palo has the hallmarks of a T7-like virulent phage, many Palo genes have homologs in alphaproteobacterial genomes; for example, the Palo *terL* gene is closely related to a gene in *Rhizobium* sp. strain Leaf341 (NCBI GenBank accession number NZ_LMPE00000000) that is part of a cluster of unannotated phage lysis, structural, and replication genes. This finding suggests that Palo may be derived from a recent temperate ancestor. In addition, Palo encodes a close homolog of A1, one of two early proteins required for second-step DNA transfer in the paradigm siphophage T5 (19).

### Data availability.

The Palo genome was deposited under GenBank accession number MT708544.1. The associated BioProject, SRA, and BioSample accession numbers are PRJNA222858, SRR11558343, and SAMN14609646, respectively.
